# A potential and novel type transgenic corn plant for control of the Corn Borer

**DOI:** 10.1038/srep44105

**Published:** 2017-03-14

**Authors:** Zhen Yue, Xiangrui Li, Enyan Zhang, Xiaoxia Liu, Zhangwu Zhao

**Affiliations:** 1 Department of Entomology, College of Plant Protection, China Agricultural University, Beijing, China; 2 State Key Laboratory for Biology of Plant Diseases and Insect Pests, Institute of Plant Protection, Chinese Academy of Agricultural Sciences, Beijing, China

## Abstract

The corn borer is a world-wide agricultural pest. In this study, a full-length neuropeptide F (*npf*) gene in *Ostrinia furnacalis* was sequenced and cloned from a cDNA library, in which the *npf* gene produces two splicing mRNA variants - *npf1* and *npf2* (with a 120 bp segment inserted into the *npf1* sequence to generate *npf2*). A spatio-temporal expression analysis showed that the highest expression level of *npf* was in the midgut of 5^th^ instar larvae (the gluttony period), and their *npf* expression and food consumption were significantly promoted after food deprivation for 6 h. When *npf* was knocked down by double-stranded RNA for NPF, larval food intake, weight and body size were effectively inhibited through changes of a biosynthesis and metabolism pathway; *i.e.* gene silencing of NPF causes decreases of total lipid and glycogen and increases of trehalose production. Moreover, we produced transgenic corn plants with stably expressed dsNPF. Results showed that *O. furnacalis* larvae fed on these transgenic leaves had lower food consumption and smaller body size compared to controls. These results indicate that NPF is important in the feeding control of *O. furnacalis* and valuable for production of potential transgenic corn.

Corn is a huge economic crop. Its planting area in China has reached 40 million hectares, and production is close to 220 million tons in 2014. The corn borer is a major corn pest, which is broadly distributed in the world and causes significant economic damage to corn, sorghum, millet, cotton, and other crops, due to its omnivorous character^1^,^2^,^3^. Genetically modified (GM) crops have been planted for several decades since the first commercialized GM crops were released in 1994^4^. Although transgenic crops producing Bt toxins for pest control have been successful^5^, it was reported that corn borer has developed resistance to the Bt corn in laboratory selection and in the field because of misuse of insecticides^6^, which also cause serious pollution of the environment. Thus, alternative ways for controlling this pest are necessary.

RNAi is a newly identified post-transcriptional mechanism in which the expression of a gene is specifically inhibited by its cognate double-stranded RNA (dsRNA). It is highly conserved among higher eukaryotes^7^,^8^. The inhibition produced by RNAi highly resembles the loss-of-function or gene knockout phenotype^9^. Previous reports showed that gene silencing in Lepidoptera insects was an important and effective tool for functional studies. When European corn borer was treated with dsRNA of chitin hydrolase, its body weight decreased 54%^10^. After treatment with low doses of dsRNA of ten target genes, the larval development of the Asian corn borer may be delayed, while dsRNA treatments with high doses cause mostly larval death^11^. Therefore, RNAi is not only a powerful tool for rapidly analyzing gene functions but also a potential method for pest control.

Neuropeptides play a central role in regulation of development, reproduction, feeding and many other physiological processes in animals^12^,^13^,^14^. The neuropeptide Y family (NPY) is one of the most widely distributed neuropeptides in the central nervous system (CNS) of vertebrates, as it is involved in modulation of many physiologies and behaviors, such as energy homeostasis, circadian rhythm, food intake, reproduction, anxiety, seizures, learning and memory, and addiction to alcohol^15^,^16^,^17^,^18^. One function of NPY is regulation of feeding behavior, in which the NPY neurons influence feeding behavior by the hypothalamus^19^. The neuropeptide F (NPF) mainly found in invertebrates is identified as a member of the NPY family^20^,^21^, because of their similar function in a signaling pathway via G protein-coupled receptors^22^. However, their peptide sequences and structures differ greatly among animal species. NPF was identified from some insect species, such as *Helicoverpa zea*^23^, *Drosophila melanogaster*^24^, *Schistocerca grearia*^25^, *Aedes aegypti*^26^, *Anopheles gambiae*^22^, *Locusta migratoria*^27^, *Bombyx mori*^20^*, Helicoverpa assulta*^28^, and *Helicoverpa armigera*^29^. However, reports of its involvement in regulating feeding are mainly from some model insects such as fruit fly and mosquito^13^,^30^, and feeding function of NPF in agricultural pests only was reported in our previous publication on *H. armigera*^29^. To explore whether NPF in *O. furnacalis* is involved in larval feeding or not, we identified and cloned a *npf* gene from *O. furnacalis*, and this was used for further functional studies. These data will serve as an important step forward to provide novel targets for the sustainable management of this pest.

## Results

### Identification and cloning of NPF

**Figure 1:**
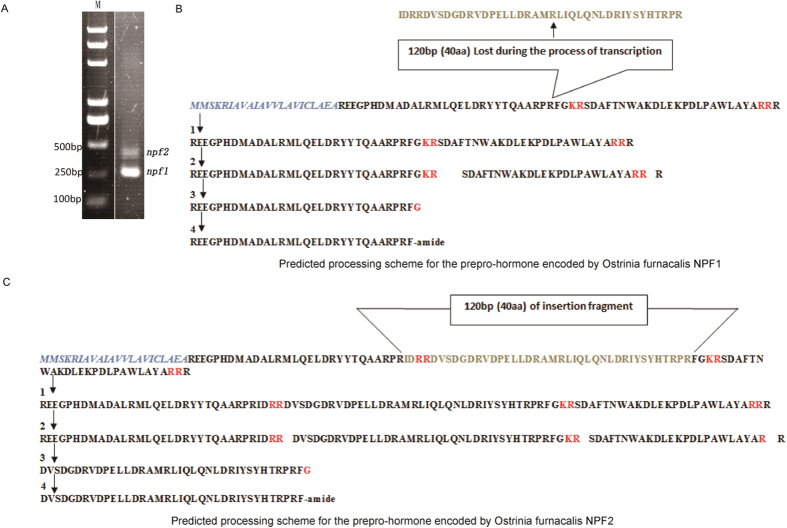
The two splicing variants of *npf* and their amino acid sequences in *O. furnacalis*. (**A**) The PCR products of *Ofurnpf1* and *Ofurnpf*2. (**B,C**) Predicted processing scheme for the amino acid sequences encoded by *Ofurnpf1 and Ofurnpf2,* respectively, in which the putative signal peptide is shown in italics. 1 represents the processing of the prepropeptides at their signal peptidase cleavage locus; 2 shows the propeptide sequences with putative propeptide convertase cleavage locus; 3 shows the putative peptides liberated via propeptide convertase with their carboxypeptidase processing sites (C’ terminal basic residues); 4 shows the mature peptides generated by carboxypeptidase with glycine residues targeted by peptidylglycine-amidating monooxygenase. 120 bp was lost from *Ofurnpf1* due to RNA splicing, not from transcription.

**Figure 2:**
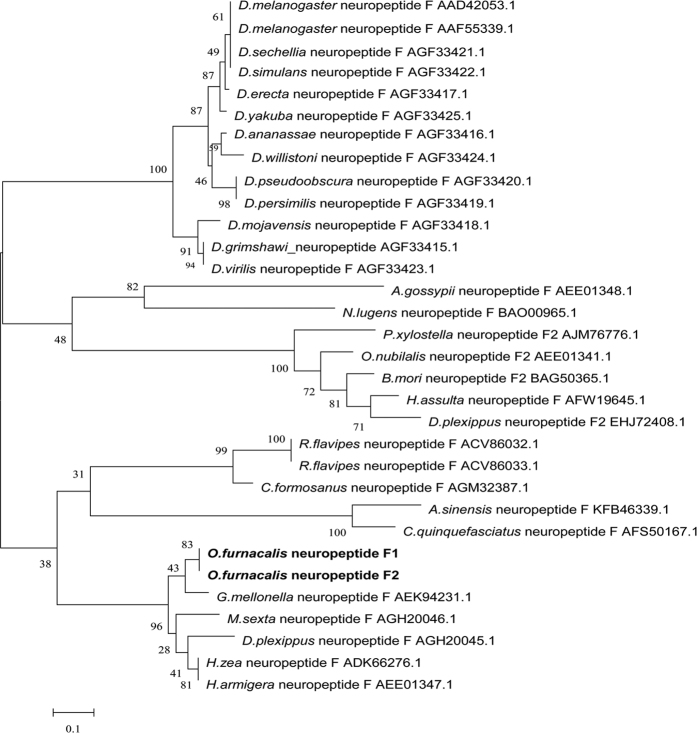
The phylogenetic relationships of insect NPF. The tree was constructed by the neighbor-joining method using MEGA software. Bootstrap analyses of 1,000 replications are shown. The sequences were obtained from GenBank. The full names of the species in phylogenetic tree are *Drosophila erecta, Drosophila yakuba, Drosophila ananassae, Drosophila willistoni, Drosophila pseudoobscura, Drosophila persimilis, Drosophila mojavensis, Drosophila grimshawi, Drosophila virilis, Aphis gossypii, Nilaparvata lugens, Plutella xylostella, Ostrinia nubilalis, Bombyx mori, Helicoverpa assulta, Danaus plexippus, Reticulitermes flavipes, Coptotermes formosanus, Anopheles sinensis, Culex quinquefasciatus, Ostrinia furnacalis, Galleria mellonella, Manduca sexta, Danaus plexippus, Helicoverpa zea, Helicoverpa armigera,* respectively.

In this study, a full-length clone of *O. furnacalis npf* was identified, isolated and cloned by the same methods as in Liu *et al*.^28^. The *npf* gene contained two splicing variants (Fig. 1A), *Ofurnpf1* and *Ofurnpf2*, with ORFs of 246 and 366 bp, respectively, in which *Ofurnpf2* is formed by inserting a 120 bp segment between the 153^th^ and 154^th^ nucleotides the *Ofurnpf1* sequence (Fig. 1B). *Ofur*NPF1 and *Ofur*NPF2 are composed of 30 and 37 amino acids, respectively. Their structures and the shearing processes producing the mature peptides are shown in Fig. 1C, in which a series of posttranslational modification were carried out by sequential action of two enzymes according to McVeigh^31^. The neighbor-joining phylogenetic trees showed that NPFs of *O. furnacalis* are closest to those of other Lepidoptera insects (Fig. 2).

### Spatio-temporal expression and function assays of *Ofurnpf*

**Figure 3:**
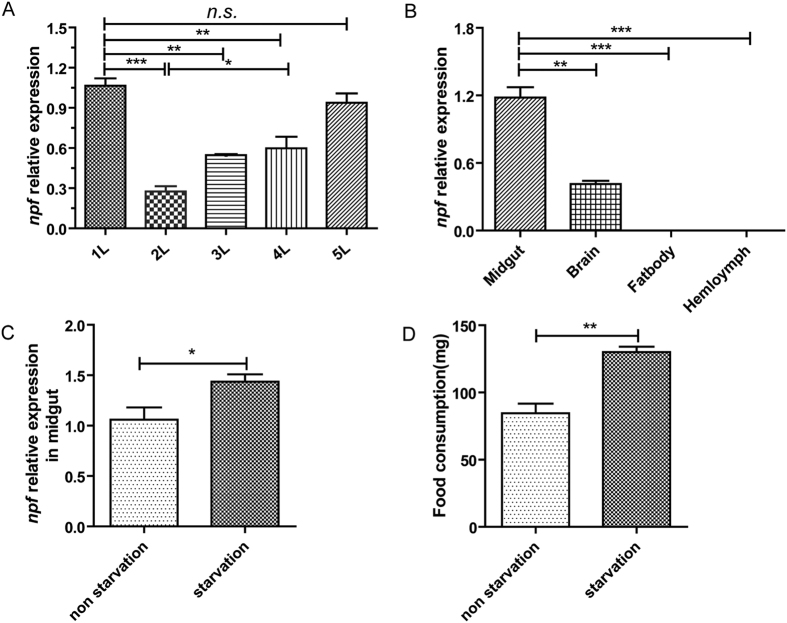
Spatio-temporal expression of larval *Ofurnpf* and effects of starvation on feeding and *Ofurnpf* level. (**A**) *Ofurnpf* expression in different larval instars. (**B**) *Ofurnpf* expression from the 5th larval instar in different tissues. (**C**) The 5th larval instar was reared with agarose (food deprivation) and normal food separately for 6 h, and the *Ofurnpf* expression in midgut was quantitatively analyzed by qRT-PCR. Each independent experiment was determined by triplicates with 30 individuals in total. (**D**) Determination of the food consumption after 6 h food deprivation, after which both starved and unstarved controls were given artificial diet for 1 h separately. Each independent experiment was determined with 20 individuals. Results were obtained from three independent biological replicates. The mean ± SD was used for expression level.

The spatio-temporal expression of *Ofurnpf* containing the two splicing variants was explored by qRT-PCR. The results showed that it was significantly higher in the 1^st^ instar larvae (just emerging as larvae from egg shells) and then decreased to the lowest point in the 2^nd^ instar larvae. Afterwards, it gradually increased from the 2^nd^ instar larvae and attained its highest level at the 5^th^ instar (the period for gluttony) (Fig. 3A). The *Ofurnpf* was mainly expressed in midgut, which exhibited significantly higher levels than other tissues (Fig. 3B). Importantly, when 5^th^ instar larvae were starved for 6 h, the *Ofurnpf* expression was significantly increased in the midgut of starved larvae compared with that in the control group fed during this period (Fig. 3C), with a rise of 26.25% compared to control (P < 0.05). Moreover, there was a significant increase of feeding amount in larvae starved prior to the feeding period (P < 0.01) (Fig. 3D). These results together suggested that *Ofurnpf* is involved in modulating feeding behavior.

### Role of NPF in energy metabolism

**Figure 4:**
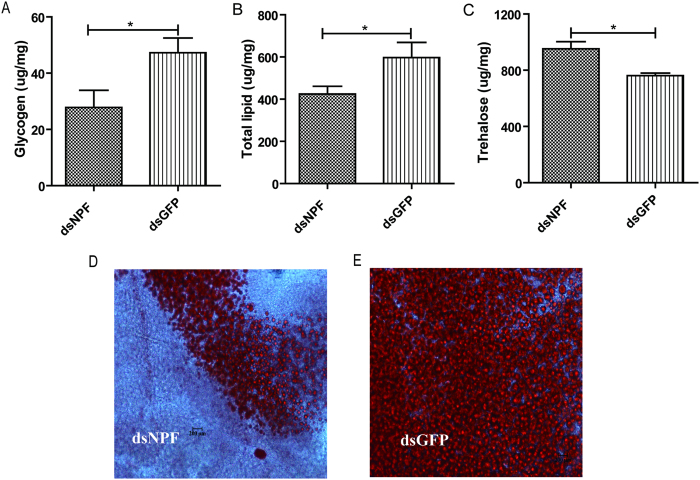
Effect of dsNPF on energy metabolism. 10 μg/3 μl of dsNPF or dsGFP was injected into the 5^th^ instar larvae of just ecdysis. Samples were collected and assayed at 72 h after injection. Whole-body homogenates were used to measure glycogen, total lipid and trehalose contents, and the fat body was used to assay lipid droplets. Glycogen (**A**) and total lipid (**B**) levels drastically decreased in dsNPF-treated larvae compared with control counterparts, and trehalose content (**C**) in dsNPF-treated larvae significantly increased (p < 0.05). Lipid droplets in dsNPF larvae (**D**) were much less than those in dsGFP control larvae (**E**). The data represent the means ± SD. Each independent experiment was performed with triplicates with 10 individuals for each repeat.

In order to further understand the regulatory mechanism of NPF on feeding, we analyzed the relationship between NPF and energy metabolism, in which glycogen, total lipid and trehalose, the main energy sources in insects, were assayed. Fifth instar larvae were injected with dsNPF or dsGFP, and then the samples were prepared and assayed after 72 h of treatment. Our results showed that glycogen and total lipid levels drastically decreased in whole body homogenates of dsNPF-treated larvae, with 41.09% (P < 0.05) and 38.89% (P < 0.05) decreases compared to control (Fig. 4A and B). On the contrary, trehalose content in dsNPF-treated larval was significantly increased, with a 20% increase compared to control (P < 0.05) (Fig. 4C). Moreover, assays of lipid droplets with Oil Red O in the fat bodies showed that the amount of lipid droplets in dsNPF larvae is much less than those in dsGFP control larvae (Fig. 4D and E). These results suggest that NPF regulates feeding behavior through a pathway of energy metabolism, in which dsNPF inhibits biosynthesis and promotes metabolism.

### Silencing and feeding effects of dsNPF

**Figure 5:**
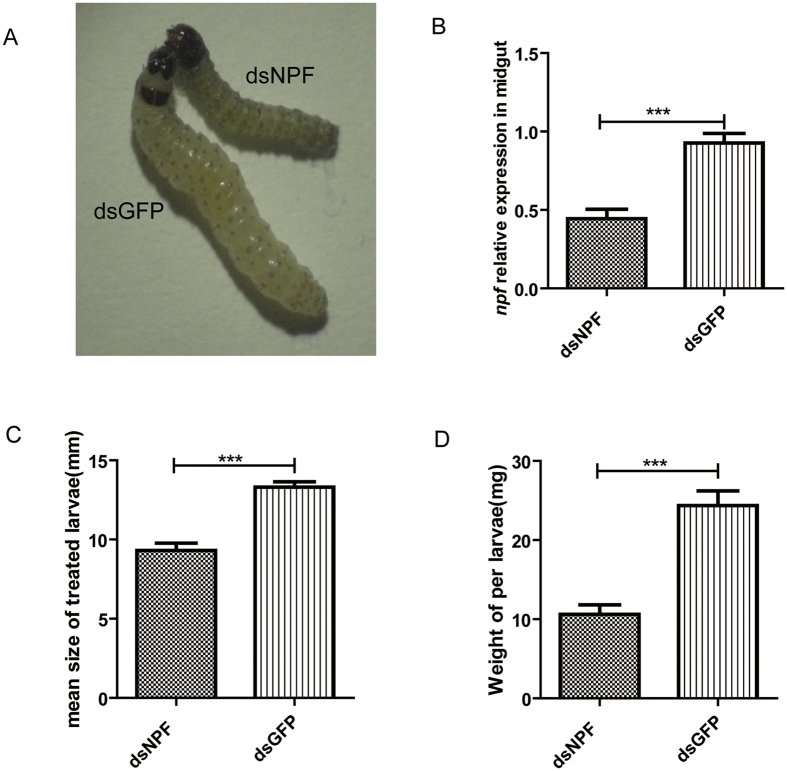
Feeding effects of silencing NFP by food consumption. (**A**) Larval phenotypes after feeding dsNPF or control dsGFP, in which the treated larvae were significantly smaller than controls. (**B**) The *npf* expression level was reduced 52% compared to control (p < 0.001). (**C** and **D**) Showed body size and body weight of larvae fed with dsNPF were decreased by 30.76% and 56.47% (respectively) compared to those fed with dsGFP (P < 0.001). The data represent the means ± SD. *P < 0.05.

We designed a feeding-based RNAi technique to repress the expression of *Ofurnpf*. Immediately after hatching from eggs, the larvae were fed on an artificial diet mixed with dsNPF, or with dsGFP as a control. After 11 days, larval midguts were dissected for assessing *npf* levels, and its effects on body weight and size of the larvae were also analyzed. Results showed that body size in the treated dsNPF larvae was significantly smaller than that in dsGFP larvae (Fig. 5A) with a reduced *npf* expression level [52% decrease compared to control (p < 0.001)] (Fig. 5B). After 11 days of feeding dsNPF, larval body size and body weight were decreased to 30.76% and 56.47%, respectively, compared to those fed on the diet containing dsGFP (P < 0.001) (Fig. 5C and D, & Table 1).

**Figure 6:**
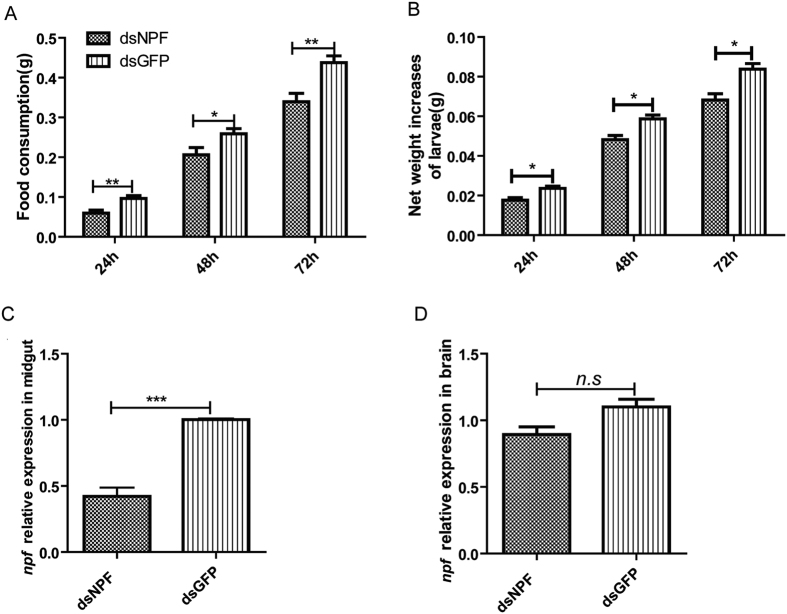
Feeding effects of silencing NPF by injection. (**A**) Food consumption at 24 h, 48 h and 72 h after treatment, respectively. (**B**) Larval net weight at 24 h, 48 h and 72 h after treatment, respectively. (**C**) Relative expression levels of *npf2* in midguts of the dsNPF- and dsGFP-injected larvae analyzed by qRT-PCR after 72 h treatments. (**D**) Relative expression levels of *npf* in brains of the dsNPF- and dsGFP-injected larvae analyzed by qRT-PCR after 72 h treatments. Each independent experiment was determined with triplicates with 28 individuals in total. The mean ± SD was used.

Furthermore, when 5^th^ instar larvae were injected with dsNPF, both food consumption and larval net weight were significantly lower than in control counterparts, with a decrease of food consumption of 38.59% (P < 0.01), 20.45% (P < 0.05) and 22.50% (P = 0.001) (Fig. 6A & Table 2) and a decrease of larval net weight of 25.11% (p < 0.01), 15.73% (p = 0.009) and 18.23% (p < 0.01), after treatments at 24 h, 48 h and 72 h (Fig. 6B & Table 2). The *npf* accumulation was significantly reduced in midgut after larvae were treated with dsNPF for 72 h, with a decrease of 57.91% compared to control (P < 0.001) (Fig. 6C), but not in brain (P > 0.05) (Fig. 6D). All these results indicate that NPF in midgut regulated feeding in *O. furnacalis*, and dsNPF is an effective tool for control feeding behavior.

### Larval feeding inhibition on transgenic maize expressing dsNPF

**Figure 7:**
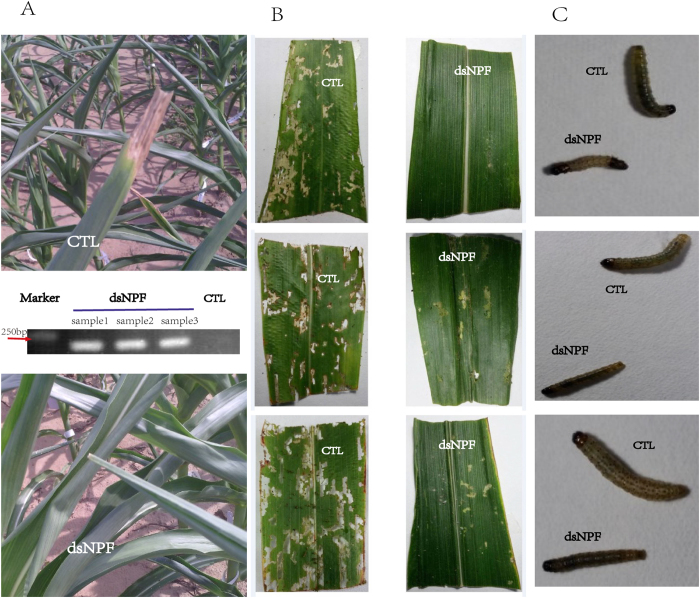
Larval feeding effects on dsNPF transgenic maize leaves. (**A**) dsNPF transgenic and non-transgenic (as control) maizes, as determined by the representative PCR analysis. (**B**) The leaves separately eaten by 5, 10 and 15 individuals/each repeat, indicated from above to below in the figure. (**C**) The larval volumes were separately determined on day 4, 7 and 11 after treatments, indicated from above to below in the figure. Each independent experiment was performed with triplicates.

The constructed dsNPF-pCAMBIA3301 plasmid was transformed into the *A. tumefaciens* strain EHA105, and the transgenic maizes were obtained through agrobacterium transient transfection for stable expression of dsNPF. Their seeds were grown until the leaf stage used for identification of the positive corn plants by PCR. The identified NPF-positive and GFP-positive transgenic corn plants were further grown (Fig. 7A). Larvae were added to the mature transgenic corn leaves and incubated in *petri* dishes (5, 10 and 15 larvae/one leaf/petri dish, assayed separately) for feeding assays. Results showed that the leaf area of the dsNPF transgenic corn eaten by larvae was much less than that of the dsGFP transgenic control (p < 0.001) (Fig. 7B). The larval body size after feeding on transgenic leaves was also significantly smaller than for the controls at day 4 after treatments (p < 0.05) (Fig. 7C & Table 3). Importantly, the *npf* RNAi results in high mortality of this insect, in which all individuals did not normally pupate or emerge because of undeveloped bodies (Table 3). All these results indicate that NPF regulates feeding behavior, and the dsNPF transgenic cotton is a potentially efficient biotechnology for field control of *O. furnacalis.*

## Discussion

In this study, we cloned an *O. furnacalis npf* gene, which has a regulatory role on feeding. The *npf* was found to have two splicing forms, with a 120 bp segment in the long *npf (npf2*) inserted into the short *npf (npf1*) sequence. A single gene encoding more than one protein was discovered in the 1980 s, and since then alternative RNA splicing has become recognized as a normal aspect of eukaryotic gene regulation^32^. In this study, we focused on the long segment of *npf* (containing both *npf*1 and *npf*2) mentioned as *npf*/NPF/dsNPF in this paper. In addition, our analysis of both isoforms herein shows expression of *npf* mainly in the midgut, where it attained its highest level at the larval gluttony stage – the 5^th^ instar. However, its expression at 1^st^ instar (just emerging into larvae from egg shells) was also higher, suggesting that feeding at 1^st^ instar larvae is also important, which was similar to that in *Helicoverpa armigera*^29^.

NPF in *D. melanogaster* was found to improve feeding regulated by the insulin signal through the InR (insulin receptor)/PI3K/S6K pathway^13^,^33^. In this study, we further found that the *O. furnacalis* NPF regulates feeding by affecting energy metabolism, in which down-regulated NPF causes decreases of glycogen and total lipid, and increases of trehalose. That is, the *O. furnacalis* NPF promotes biosynthesis or energy storage and inhibits metabolism or energy utilization. These results suggest that insulin signal may regulate energy metabolism through the NPF system, which is a hypothesis for further analysis.

Terenius reported that the majority of RNAi studies in Lepidoptera were from *Bombyx mori, Manduca sexta* and some Noctuidae species^34^. Systemic RNAi has been demonstrated in some species, such as *Hyalophora cecropia* and *B. mori*, in which injection of dsRNA into the pupa can result in phenotypic effects in developing embryos. However, a great variation of sensitivity to systemic RNAi has been seen among different Lepidopteran species^34^. Therefore, it is important to determine the sensitivity to RNAi for each species.

It has been reported that either injection or feeding is a feasible way to deliver dsRNA into several Lepidopteran insects to produce an RNAi effect, including *Spodoptera exigua*^35^*, Helicoverpa assilta*^28^, *Ostrinia nubilalis*^10^ and *Helicoverpa armigera*^29^. In these cases, the injected dsRNA causing the interfering effect might be cut into siRNA by DICER. For field control of pests, transgenic plants engineered to express insect dsRNAs have been reported^36^,^37^. In this study we knocked NPF down in larvae by injections and feeding of dsNPF, as well as by feeding the larvae transgenic corns stably expressing dsNPF, and the results showed that all these methods could effectively suppress the expression of *Ofurnpf* and feeding behavior. Therefore, NPF is important for growth and development of *O. furnacalis* and could be used as a target gene for plant protection, with the further goal to explore transgenic dsNPF-producing corn plants for control of pest insects.

## Materials and Methods

### Insect rearing

The eggs of *O. furnacalis* were obtained from Dr. Zhenying Wang’s lab (Chinese Academy of Agricultural Sciences). After hatching, larvae were reared in boxes (20 × 14 × 8 cm^3^) using an artificial diet (maize flour 150.0 g, soybean flour 150.0 g, glucose 75.0 g, vitamin C 4.0 g, agar 22.0 g, yeast power 90.0 g, sorbic acid 5.0 g, propionic acid 2.0 mL, water 1400 mL), while adults were fed with 5% honey water and laid eggs on waxed papers. All stages of *O. furnacalis* were kept at 25 ± 1°C and 65% relative humidity under a photoperiod of 16 L: 8D.

### RNA extraction and cDNA library construction

Total RNA from each female or male sample was extracted, purified and checked on 1% agarose gels, and further integrity was also confirmed using the 2100 Bioanalyzer (Agilent Technologies). The specific methodology was same as the reference described by Liu *et al*.^28^.

### *npf* cloning

Total RNA was extracted from the 5^th^ instar larvae of *O. furnacalis* using TRIzol Reagent (Tiangen, Beijing, China), and 1 μg RNA was used to synthesize the first-strand cDNA using a Fast Quant RT Kit (Tiangen, Beijing, China). NPF coding sequence was amplified from *O. furnacalis* cDNA by PCR with primers (NPF F & R) (Supplementary Table 1). PCR products were run on 1.5% agarose gels and stained with ethidium bromide. PCR products were purified using a Universal DNA Purification Kit (Tiangen, Beijing, China). The purified fragments were cloned into the PMD19-T Simple vector and transformed into *Escherichia coli* DH5α cells. The clone was sequenced using primers M13f & M13r. The positive clones were sequenced to verify the correct size and sequence of inserts. The *O. furnacalis* NPF signal peptides were predicted by refer to the description by Liu *et al*.^28^.

### Spatio-temporal expression of *npf*

To determine the expression level of *npf* in *O. furnacalis*, the *npf* levels in different larval stages and tissues were examined by real-time quantitative reverse transcription polymerase chain reaction (qRT-PCR). Larvae were collected at the second day of every instar, and their total RNA was extracted for analysis of temporal expression. For analysis of spatial expression of *npf*, the brain (Br), midgut (MG), fat body (FB) and hemloymph (Hem) from 5^th^ instar larvae were collected. cDNA from comparable reverse-transcription reaction was used for qPCR on the ABI Stepone (Applied Biosystem, Foster, CA, USA). The RpL8 gene was amplified as an internal reference with a stable expression in different developmental stages. Relative expression of the targets gene in different stages and tissues was conducted according to threshold cycle (Ct) value based on the 2^−ΔΔCT^ method. All tests were done with triplicates.

### Effects of food deprivation on feeding and *npf* level

After 24 h of normal feeding, 5^th^ instar larvae were reared with agarose (food deprivation) and normal food separately. The larvae were collected for quantitative analysis of *npf* expression and feeding after food deprivation for 6 h. The qRT-PCR method and analysis were the same as above, and the feeding assays were the same as in a previous report^29^. The experiments were explored with triplicates independently, with 10 individuals at each repeat.

### Determination of total lipid, glycogen and trehalose

Whole-body homogenates of each individual were used to extract glycogen, trehalose and total lipid, and these were detected as previously described^29^.

### Variation of lipid droplets in fat body

The just ecdysis of fifth-instar larvae were treated with dsNPF or dsGFP (method same as above). Each group was performed in triplicates with 3 individuals in each repeat (n = 9 larvae). Both controls and treatments were reared with a certain amount of artificial diets renewed every day. After 72 h of normal feeding, the fat bodies were dissected in PBS for assays of lipid droplets. The lipid tissues were fixed in 4% paraformaldehyde/PBS for 30 min at room temperature. After that tissues were rinsed three times with 1 × PBS, incubated for 40 min in 0.06% Oil Red O (Sigma), and then rinsed three times with 1 × PBS. Staining samples were mounted in 75% glycerol. All images were taken using a Nikon confocal microscope.

### dsRNA synthesis

dsRNA was synthesized using the T7 RiboMAX™ Express RNAi System and protocols (Promega, USA). Purified dsRNAs were quantified by spectroscopy and examined by agarose gel electrophoresis to ensure their integrity. The NPF coding fragment of 399 bp was selected as an RNAi target-sequence. PCR primers with T7 promoter sequences were used to prepare double-stranded RNA (Supplementary Table 1). The primers for green fluorescent protein gene (GFP) as a control are also shown in Supplementary Table 1. PCR products were purified and sequenced.

### Application of dsRNA by feeding artificial food

Immediately after they hatched from eggs, larvae were individually fed on fresh artificial diet containing dsNPF or dsGFP (as a control), in which each larvae was reared with 24 μg dsNPF or dsGFP. On day 11, the larval body sizes were examined separately for each condition. The experiments were performed with triplicates with 10 individuals in each repeat. The details of treatments are listed in Supplementary Table 2.

### Application of dsRNA by injection

Double-stranded RNAs were injected into fifth-instar larvae to investigate their effects on food consumption. 3 μl of dsRNA(10 μg) were injected into the lateral intersegmental membrane between the third and fourth abdominal segment. Samples included the treatment groups injected with dsNPF and control groups injected with an equivalent volume of dsGFP. Each group was performed in triplicates with 10 individuals at each repeat (n = 30 larvae/group). Both controls and treatments were reared with a certain amount of artificial diets refreshed every day. After 24/48/72 hours, larval weight, the remainder of the artificial diet and the feces were observed separately. As food is fresh initially, it is also necessary to set a blank experiment as a control, measuring the weight change of diet caused by water volatilization. Larval food consumption is calculated by same method as previously described^27^. The accumulation of *npf* mRNA after dsRNA treatment was also investigated by qRT-PCR, which was accomplished as above.

### Construction of plasmids

Plasmids were constructed using standard cloning techniques. dsRNAi constructs were prepared by adding appropriate restriction sites to the ends of the primers used to perform PCR amplification with DNA polymerase (TIANGEN) and primers (p-NPF-F and p-NPF-R) in Supplementary Table 1. The PCR reactions began with 94 °C denaturation for 3 min, then 35 cycles of denaturation at 94 °C for 30 s, 55 °C annealing for 30 s, and 72 °C extension for 1 min. The PCR products and pCAMBIA3301 vector were digested separately with restriction enzymes BstEII and NcoI (TAKARA Co.). The restriction sites of dsNPF were between the CaMV 35 s promoter and Nos Poly A elements of pCAMBIA3301 vector, shown in supplementary Figure 1. They then were further purified, ligated and transformed into DH5a. The newly constructed plasmid was named dsNPF-pCAMBIA3301 plasmid. The control plasmid dsGFP-pCAMBIA3301 was constructed with the same method described above, using primers for dsGFP.

### Maize transformation and larval feeding

Each binary expression vector was transformed into *A. tumefaciens* strain EHA105, which was subsequently used to infect immature maize embryos. The specific method for maize transformation is the same as the one used by Zhu *et al*.^38^. Genomic DNA from each transformed corn identified positive strains by PCR.

For larvae feeding on corn leaves in the lab, the transgenic corn leaf was then placed in a petri dish with a moist filter paper and the 3^rd^ instar larvae were allowed to feed on the leaf. After every 24 h, the leaf area eaten was measured. The treatment was performed in triplicates with 5, 10 and 15 larval individuals assayed separately for each repeat.

### Data analysis

All statistical analysis was conducted using GraphPad Prism 5. Two groups of data were analyzed with a 2-tailed, unpaired t-test. More than 2 groups of data were analyzed with one-way ANOVA followed by the Tukey-Kramer HSD Test as the post hoc test.

## Additional Information

**How to cite this article:** Yue, Z. *et al*. A potential and novel type transgenic corn plant for control of the Corn Borer. *Sci. Rep.* **7**, 44105; doi: 10.1038/srep44105 (2017).

**Publisher's note:** Springer Nature remains neutral with regard to jurisdictional claims in published maps and institutional affiliations.

## Supplementary Material

Supplementary Information

## Figures and Tables

**Table 1:** Mean size and mean weight of larvae fed dsNPF.

Treatment stage	treatment	ds NPF n = 20	ds GFP n = 22	P value	decrease%
First instar larvae	NPF relative expression	0.4443 ± 0.0596	0.9265 ± 0.0619	0.001	52.05%
	Mean size (mm)	9.300 ± 0.464	13.432 ± 0.245	<0.001	30.76%
	Mean weight (mg)	10.610 ± 1.202	24.373 ± 1.833	<0.001	56.47%

**Table 2:** Feeding effects of silencing NFP by injection.

Larval Number		dsNPF	dsGFP	P value	decrease%
NPF relative expression (n = 9)	midgut	0.42 ± 0.07	1.00 ± 0.01	0.0001	57.91%
	brain	0.89 ± 0.06	1.10 ± 0.06	0.064	no significance
Food consumption(mg) (n = 28)	24 h	5.92 ± 0.80	9.64 ± 0.71	0.002	38.59%
	48 h	20.62 ± 1.83	25.92 ± 0.13	0.021	20.45%
	72 h	33.92 ± 2.14	43.77 ± 0.17	0.001	22.50%
Larval net weight(mg) (n = 28)	24 h	1.76 ± 0.14	2.35 ± 0.15	0.006	25.11%
	48 h	4.82 ± 0.21	5.72 ± 0.25	0.009	15.73%
	72 h	6.82 ± 0.32	8.34 ± 0.34	0.002	18.23%

**Table 3:** Impacts on growth and mortality rate of larvae fed dsNPF transgenic corn leaves.

Larval Number	Days After treatments	Larval instar	Survival Number (Mortality rate %)		Body Size (mm)		P value
			control	Treatment	control	treatment	
Feeding of 5 larvae in each repeat with triplicates (n = 15)	1	3	15(0)	15(0)	14.0 ± 0.10	13.8 ± 0.15	−
	4	3	15(0)	4(73)	16.9 ± 0.11	16.0 ± 0.08	P < 0.05
	7	4	13(13)	0(100)	20.3 ± 0.13	−	−
	11	4	10(33)	−	24.9 ± 0.07	−	−
Feeding of 10 larvae in each repeat with triplicates (n = 30)	1	3	30(0)	30(0)	13.6 ± 0.09	13.8 ± 0.08	−
	4	3	29(1)	19(37)	17.9 ± 0.13	16.8 ± 0.11	P < 0.05
	7	4	24(20)	8(73)	22.3 ± 0.18	17.5 ± 0.13	P < 0.01
	11	4	23(23)	0(100)	25.4 ± 0.08	−	−
Feeding of 15 larvae in each repeat with triplicates (n = 45)	1	3	45(0)	45(0)	14.0 ± 0.09	13.9 ± 0.09	
	4	3	38(15)	22(51)	17.7 ± 0.11	16.3 ± 0.08	P = 0.0572
	7	4	34(24)	12(73)	21.8 ± 0.15	17.2 ± 0.12	P < 0.01
	11	4	29(35)	0(100)	25.6 ± 0.10	*−*	−

## References

[b1] Wang Z., Lu X., He K. & Zhou D. Review of history, present situation and prospect of the Asian maize borer research in China. J. Shenyang Agr. Univ. 31, 402–412 (1999).

[b2] Afidchao M. M., Musters C. & de Snoo G. R. Asian corn borer (ACB) and non‐ACB pests in GM corn (Zea mays L.) in the Philippines. Pest Manag. Sci. 69, 792–801 (2013). 23401215 10.1002/ps.3471

[b3] Li J. . The genetic structure of Asian corn borer, *Ostrinia furnacalis*, populations in China: Haplotype variance in Northern populations and potential impact on management of resistance to transgenic maize. J. Heredity 105, 346–357 (2014). 10.1093/jhered/esu036 25024271

[b4] James C. & Krattiger A. F. Global review of the field testing and commercialization of transgenic plants: 1986 to 1995. ISAAA Briefs 1 (1996).

[b5] Thompson G. D., Dalmacio S., Criador I. V. A., Alvarez E. & Hechanova R. Field performance of TC1507 transgenic corn hybrids against Asian corn borer in the Philippines. Phil. Agr. Sci. 93, 375–383 (2011).

[b6] Xu L. N. . Transcriptome differences between Cry1Ab resistant and susceptible strains of Asian corn borer. BMC Genomics 16, 1–15 (2015). 25886725 10.1186/s12864-015-1362-2 PMC4406038

[b7] Carthew R. W. & Sontheimer E. J. Origins and mechanisms of miRNAs and siRNAs. Cell 136, 642–655 (2009). 19239886 10.1016/j.cell.2009.01.035 PMC2675692

[b8] Berezikov E. Evolution of microRNA diversity and regulation in animals. Nat. Rev. Genetics 12, 846–860 (2011). 22094948 10.1038/nrg3079

[b9] Hammond S. M., Bernstein E., Beach D. & Hannon G. J. An RNA-directed nuclease mediates post-transcriptional gene silencing in *Drosophila* cells. Nat. 404, 293–296 (2000). 10.1038/35005107 10749213

[b10] Khajuria C., Buschman L. L., Chen M. S., Muthukrishnan S. & Zhu K. Y. A gut-specific chitinase gene essential for regulation of chitin content of peritrophic matrix and growth of *Ostrinia nubilalis* larvae. Insect Biochem. Molec. Biol. 40, 621–629 (2010). 20542114 10.1016/j.ibmb.2010.06.003

[b11] Wang Y., Zhang H., Li H. & Miao X. Second-generation sequencing supply an effective way to screen RNAi targets in large scale for potential application in pest insect control. PloS one 6, e18644 (2011). 21494551 10.1371/journal.pone.0018644 PMC3073972

[b12] Gäde G. Regulation of intermediary metabolism and water balance of insects by neuropeptides. Ann. Rev. Entomol. 49, 93–113 (2004). 14651458 10.1146/annurev.ento.49.061802.123354

[b13] Wu Q., Zhao Z. & Shen P. Regulation of aversion to noxious food by *Drosophila* neuropeptide Y–and insulin-like systems. Nat.Neurosci. 8, 1350–1355 (2005). 16172603 10.1038/nn1540

[b14] Van Wielendaele P. . Neuropeptide F regulates male reproductive processes in the desert locust, Schistocerca gregaria. Insect biochem. Molec. Biol. 43, 252–259 (2013). 23295785 10.1016/j.ibmb.2012.12.004

[b15] Herzog H. Neuropeptide Y and energy homeostasis: insights from Y receptor knockout models. Eur J Pharmacol. 480, 21–29 (2003). 14623347 10.1016/j.ejphar.2003.08.089

[b16] Pedrazzini T., Pralong F. & Grouzmann E. Neuropeptide Y: the universal soldier. Cell Mol Life Sci. 60, 350–377 (2003). 12678499 10.1007/s000180300029 PMC11138533

[b17] Thorsell A. & Heilig M. Diverse functions of neuropeptide Y revealed using genetically modified animals. Neuropeptides. 36, 182–193 (2002). 12359508 10.1054/npep.2002.0897

[b18] Vezzani A., Sperk G. & Colmers W. F. Neuropeptide Y: emerging evidence for a functional role in Seizure modulation. Trends Neurosci. 22, 25–30 (1999). 10088996 10.1016/s0166-2236(98)01284-3

[b19] van Swieten M. M., Pandit R., Adan R. A. & van der Plasse G. The neuroanatomical function of leptin in the hypothalamus. J Chem Neuroanat. 61-62, 207–220 (2014). 25007719 10.1016/j.jchemneu.2014.05.004

[b20] Roller L. . The unique evolution of neuropeptide genes in the silkworm *Bombyx mori*. Insect biochem. Molec. Biol. 38, 1147–1157 (2008). 19280707 10.1016/j.ibmb.2008.04.009

[b21] Huang Y., Crim J. W., Nuss A. B. & Brown M. R. Neuropeptide F and the corn earworm, *Helicoverpa zea*: A midgut peptide revisited. Peptides 32, 483–492 (2011). 20869419 10.1016/j.peptides.2010.09.014

[b22] Garczynski S. F., Crim J. W. & Brown M. R. Characterization of neuropeptide F and its receptor from the African malaria mosquito, *Anopheles gambiae*. Peptides 26, 99–107 (2005). 15626509 10.1016/j.peptides.2004.07.014

[b23] Huang Y., Brown M. R., Lee T. D. & Crim J. W. RF-amide peptides isolated from the midgut of the corn earworm, *Helicoverpa zea*, resemble pancreatic polypeptide. Insect biochem. Molec. Biol. 28, 345–356 (1998). 9692236 10.1016/s0965-1748(98)00007-1

[b24] Brown M. R. . Identification of a *Drosophila* brain-gut peptide related to the neuropeptide Y family. Peptides 20, 1035–1042 (1999). 10499420 10.1016/s0196-9781(99)00097-2

[b25] De Loof A. . Gonadotropins in insects: an overview. Arch.Insect Biochem. Physiol. 47, 29–138 (2001). 11418931 10.1002/arch.1044

[b26] Stanek D. M., Pohl J., Crim J. W. & Brown M. R. Neuropeptide F and its expression in the yellow fever mosquito, *Aedes aegypti*. Peptides 23, 1367–1378 (2002). 12182937 10.1016/s0196-9781(02)00074-8

[b27] Clynen E., Huybrechts J., Verleyen P., De Loof A. & Schoofs L. Annotation of novel neuropeptide precursors in the migratory locust based on transcript screening of a public EST database and mass spectrometry. BMC genomics 7, 537–538 (2006). 10.1186/1471-2164-7-201 PMC1574313 16899111

[b28] Liu X. G., Zhang Y. F., Zhou Z. J. & Zhao Z. W. Clonging and sequence analysis of neuropeptide F from the oriental tobacco budworm *Helicoverpa assulta* (GUENÉE). Arch. Insect Biochem. *Physiol*. 84, 115–129 (2013). 24105726 10.1002/arch.21119

[b29] Yue Z. . Development of a novel-type transgenic cotton plant for control of cotton bollworm. Plant Biotechnol. J. 14, 1747–1755 (2016). 26841044 10.1111/pbi.12534 PMC5067616

[b30] Lingo P. R., Zhao Z. & Shen P. Co-regulation of cold-resistant food acquisition by insulin-and neuropeptide Y-like systems in *Drosophila melanogaster*. Neurosci. 148, 371–374 (2007). 10.1016/j.neuroscience.2007.06.010 PMC2837932 17658221

[b31] McVeigh P., Kimber M., Novozhilova E. & Day T. Neuropeptide signalling systems in flatworms. Parasitol. 131, S41–S55 (2005). 10.1017/S0031182005008851 16569292

[b32] Leff S. E. & Rosenfeld M. G. Complex transcriptional units: diversity in gene expression by alternative RNA processing. Ann. Rev. Biochem. 55, 1091–1117 (1986). 3017190 10.1146/annurev.bi.55.070186.005303

[b33] Van der Heide L. P., Ramakers G. M. & Smidt M. P. Insulin signaling in the central nervous system: learning to survive. Progr. Neurobiol. 79, 205–221 (2006). 10.1016/j.pneurobio.2006.06.003 16916571

[b34] Terenius O. . RNA interference in Lepidoptera: an overview of successful and unsuccessful studies and implications for experimental design. J. Insect Physiol. 57, 231–245 (2011). 21078327 10.1016/j.jinsphys.2010.11.006

[b35] Chen X. . Disruption of Spodoptera exigua larval development by silencing chitin synthase gene A with RNA interference. Bull.Entomol. Res. 98, 613–619 (2008). 18662430 10.1017/S0007485308005932

[b36] Baum J. A. . Control of coleopteran insect pests through RNA interference. Nat. Biotechnol. 25, 1322–1326 (2007). 17982443 10.1038/nbt1359

[b37] Zhu J. Q. . Improvement of Pest Resistance in Transgenic Tobacco Plants Expressing dsRNA of an Insect-Associated Gene EcR. PloS one 7, e38572 (2012). 22685585 10.1371/journal.pone.0038572 PMC3369839

[b38] Zhu J. J. . Efficiency and inheritance of targeted mutagenesis in maize using crispr-cas9. J. Genet. Genom. 43, 25–36 (2016). 10.1016/j.jgg.2015.10.006 26842991

