# Supplementary material for: A potential and novel type transgenic corn plant for control of the Corn Borer
Source: Sci Rep. 2017 Mar 14;7:44105. doi: 10.1038/srep44105 (PMC5349583; doi:10.1038/srep44105)
Supplement: Supplementary Information [file srep44105-s1.pdf]

A potential and novel type transgenic corn plant for control of the Corn Borer

Zhen Yue[1#], Xiangrui Li[2#], Enyan Zhang[1], Xiaoxia Liu[1], Zhangwu Zhao[1*]

[1] Department of Entomology, College of Plant Protection, China Agricultural University

[2] State Key Laboratory for Biology of Plant Diseases and Insect Pests, Institute of Plant Protection, Chinese Academy of Agricultural Sciences

Supplementary information:

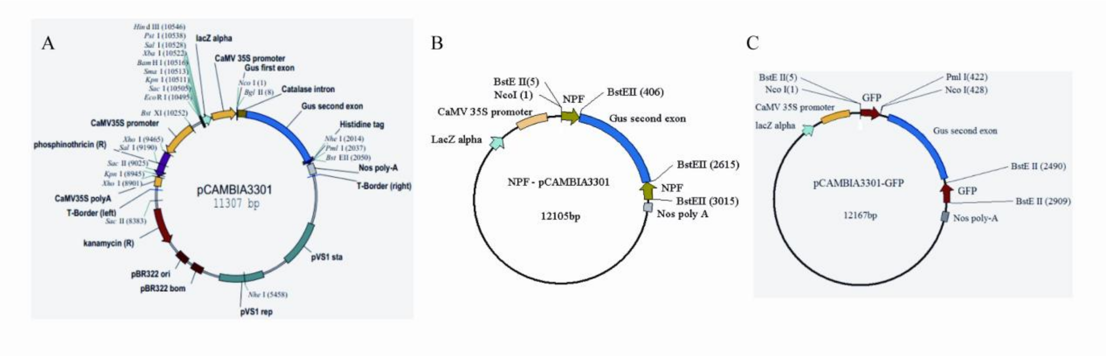

Supplementary Figure 1 Construction of dsNPF and dsGFP plasmids

Supplementary Table 1 Primers used in this paper

Application of primers	sequence	product size(bp)
NPF1 & 2	F:CGTTCCGCACATCTATC	NPF1: 279
	R:CGCCCCTCATCTCCTT	NPF2: 399
dsNPF	F:TAATACGACTCACTATAGGCGTTCCGCACATCTATC	437
	R:TAATACGACTCACTATAGGCGCCCCTCATCTCCTT	
M13	F: GTTTTCCCAGTCACGAC	PMD19T vector
	R:CAGGAAACAGCTATGAC	sequence 130bp
ACTIN	F:ACGGAGGTGGTAACCATCAACA	
RPL8	R:ACGCCTCCTTCTTGGTGTCG	

Supplementary Table 2 Treatment of feeding dsRNA

Larval state	Artificial diet	Day	dsNPF2	dsGFP
1st instar	14mg	1、2	2μg	2μg
2nd instar	16mg	3	2μg	2μg
2nd instar	20mg	4	4μg	4μg
2 or 3L	20mg	5	4μg	4μg
3th instar	22mg	6	4μg	4μg
3th instar	22mg	7	4μg	4μg
4th instar	22mg	8	4μg	4μg
4th instar	200mg	9、10	0	0
4th instar		11	Test RNA expression	
			Total: 24μg dsRNA per larvae	
